# An interpretable data-driven prediction model to anticipate scoliosis in spinal muscular atrophy in the era of (gene-) therapies

**DOI:** 10.1038/s41598-024-62720-w

**Published:** 2024-05-23

**Authors:** Tu-Lan Vu-Han, Rodrigo Bermudez Schettino, Claudia Weiß, Carsten Perka, Tobias Winkler, Vikram Sunkara, Matthias Pumberger

**Affiliations:** 1 Center for Musculoskeletal Surgery, Charité – Universitätsmedizin Berlin, corporate member of Freie Universität Berlin and Humboldt-Universität Zu Berlin, Berlin, Germany; 2 Berlin Institute of Health at Charité – Universitätsmedizin Berlin, BIH Biomedical Innovation Academy, Charitéplatz 1, 10117 Berlin, Germany; 3 Center for Humans and Machines, Max Planck Institute for Human Development, Lentzeallee 94, 14195 Berlin, Germany; 4 Department of Pediatric Neurology, Center for Chronically Sick Children, Charité-Universitätsmedizin Berlin, Augustenburger Platz 1, Berlin, Germany; 5 Institute of Health, Berlin Institute of Health Center for Regenerative Therapies, Berlin, Germany; 6 Berlin Institute of Health at Charité – Universitätsmedizin Berlin, Julius Wolff Institute, Berlin, Germany; 7 Explainable AI for Biology, Zuse Institute Berlin, Takustraße 7, 14195 Berlin, Germany

**Keywords:** Neurological disorders, Surgery, Computational models, Data processing, Machine learning, Predictive medicine

## Abstract

5q-spinal muscular atrophy (SMA) is a neuromuscular disorder (NMD) that has become one of the first 5% treatable rare diseases. The efficacy of new SMA therapies is creating a dynamic SMA patient landscape, where disease progression and scoliosis development play a central role, however, remain difficult to anticipate. New approaches to anticipate disease progression and associated sequelae will be needed to continuously provide these patients the best standard of care. Here we developed an interpretable machine learning (ML) model that can function as an assistive tool in the anticipation of SMA-associated scoliosis based on disease progression markers. We collected longitudinal data from 86 genetically confirmed SMA patients. We selected six features routinely assessed over time to train a random forest classifier. The model achieved a mean accuracy of 0.77 (SD 0.2) and an average ROC AUC of 0.85 (SD 0.17). For class 1 ‘scoliosis’ the average precision was 0.84 (SD 0.11), recall 0.89 (SD 0.22), F1-score of 0.85 (SD 0.17), respectively. Our trained model could predict scoliosis using selected disease progression markers and was consistent with the radiological measurements. During post validation, the model could predict scoliosis in patients who were unseen during training. We also demonstrate that rare disease data sets can be wrangled to build predictive ML models. Interpretable ML models can function as assistive tools in a changing disease landscape and have the potential to democratize expertise that is otherwise clustered at specialized centers.

## Introduction

### SMA and disease-modifying therapies

New (gene-)therapies are changing the natural course of diseases in a growing number of neuromuscular disorders (NMD). The first to undergo a drug-induced revolution was 5q-spinal muscular atrophy (SMA), a rare monogenic NMD with an incidence of 1:7000^1^ that is now included in a growing number of newborn screening programs worldwide^2^. The currently approved disease-modifying therapies for SMA include gene therapy onasemnogene abeparvovec as well as antisense oligonucleotides nusinersen and small molecule splicing modifier risdiplam^3–6^. Gene therapy onasemnogene uses an adeno-associated virus 9 AAV9 to introduce the complementary DNA of the *SMN1* gene encoding for the missing SMN protein. Antisense oligonucleotides and RNA targeting small molecules, on the other hand, leverage the *SMN2* pre-mRNA splicing mechanism to include exon 7, which is missing in defective SMN proteins^6,7^. The introduction of these therapies has marked a paradigm shift in previous SMA-associated orthopedic management strategies, especially for severely affected patients, such as SMA type 1, who historically rarely survived the age of two^8,9^. Up to 90% of patients develop SMA-associated neuromuscular scoliosis (NMS)^10^, which belongs to the group of flaccid NMS, characterized by rapid progression upon onset. The guiding principles of NMS management are closely linked to the progression of the underlying disease, in which the anticipation of curve and disease progression is a key to successful treatment^11^. SMA patients are now achieving motor milestones beyond their historical counterparts^12^, and the conclusions drawn from historical data sets have limited applicability. As a rare disease, the treatment of SMA requires expertise that is regularly clustered at specialized centers, where patients are evaluated at regular intervals to monitor disease progression over the course of many years. In a changing disease landscape, data-driven machine learning (ML) algorithms harbor great potential and can serve as assistive tools during patient evaluations. However, the black-box nature of ML algorithms may limit adoption by physicians during clinical decision-making^13^. A clinical support tool should be trained on clinically relevant features and interpretable to a physician. The aim of this study was to develop an interpretable data-driven ML model capable of predicting scoliosis based on the clinical features that are assessed during routine visits of SMA patients. We used explainable AI (XAI) tools to test model interpretability.

## Material and methods

### Data collection

Data was collected from genetically confirmed 5q-SMA patients, who had received SMA therapies, using REDCap electronic data capture tools^14,15^. Data was extracted from electronic health records (EHR) and Picture Archiving and Communication System (PACS). REDCap instruments included pseudonymized demographic data, SMA genetic and disease markers, clinical parameters from examinations and assessments during routine hospital visits. Motor scores were tested by physiotherapists trained in the SMArtCare registry^16^.

### Data export and preparation

The SMA data set was fully updated and exported in May 2023. The descriptive statistics of the data set and visualization was performed using pandas, Matplotlib and Seaborn packages^17–19^ implemented in Python (version 3.11). The processed data set was segmented into subsets of routine SMA visits, SMA therapy administrations and spine examinations. We performed expert domain-knowledge directed feature engineering. In summary, the variables related to a feature were aggregated and converted to numeric using current domain-knowledge of the feature. The features used for training in this study include ‘orthosis’, ‘ventilation’, and ‘contractures’ (see also Appendix).

### Feature engineering

To overcome the challenge of training a machine learning model on a small data set, we engineered features to improve the predictive power of individual features. To do this, we used expert domain knowledge from clinicians specializing in treating SMA patients and the current literature. Contractures are common sequelae in SMA patients. The contractures are routinely measured by physicians during routine visits and the anatomical location (feet, knees, hips, elbows, wrists) and severity (none, mild, moderate, severe) is documented in the patient’s EHR. According to the current literature, contractures in the lower extremities occur early during SMA disease progression and have a significant impact on the patient’s functional capabilities. To calculate the score, we ranked the location of contractures by their significance for SMA disease progression and exponentiated it with the severity of the contracture. To engineer the orthosis score, we similarly ranked the location of the orthosis by their correlation with SMA severity and converted that representation to numeric. This allowed us to numerically encode whether a patient had one or a combination of multiple different orthosis types, which indirectly correlate with SMA severity. For the ventilation score, the ventilation type (none, non-invasive, invasive), frequency of use (daily, occasional, or when ill only), and duration of use (intermittent, night, day, or continuous) were used to calculate a score that represented the intensity of the patients’s ventilation requirements.

### Feature importance testing and feature selection

For univariate feature importance analysis we performed correlation and predictive power score analysis^20^. To minimize the risk of data leakage during training, we removed all variables related to the spine. For multivariate feature importance analysis, we performed feature importance testing using mean decrease impurity analysis, permutation feature importance and Shapley Additive exPlanations (SHAP). We selected six SMA progression markers from different clinical domains to train a Random Forest Classifier (RFC). These progression markers included ‘age at assessment’, ‘CHOP-INTEND score’, ‘contractures score’, ‘HFMSE score’, ‘orthoses score’ and the ‘ventilation score’.

### Scoliosis labels and weight

Target labels for a binary classifier, class 0 = ‘no scoliosis’ and class 1 = ‘scoliosis’ were collected with the following prioritization from PACS and patients’ EHR: If anteroposterior spine radiographs were available, the Cobb angle was measured, and the scoliosis label was ‘positive’ (1) if a Cobb angle > 10° was measured. If there was no spine radiograph available, the label was collected from documented external orthopedic treatment (e.g., external radiological or orthopedic report). If neither were available, the scoliosis label was collected from clinical examinations of the spine. The scoliosis label was ‘unknown’, and the patient was excluded from the train/test data set when none of the above documentation were available. The diagnosis of scoliosis onset is marked with diagnostic uncertainty near the threshold of a Cobb angle of 10°, as is the case with any diagnostic tool. To include the uncertainty in the training a binary classifier, we incorporated weights to regulate penalization during training. Scoliosis labels of ‘0’ = ‘no scoliosis’ and ‘1’ = ‘scoliosis’, were annotated with scoliosis confidence levels, depending on the mode of acquisition (i.e., spine radiograph or clinical exam) and the severity of scoliosis (i.e., Cobb angle).

### Missing data analysis

To fine-tune the model, we performed missing data analysis using missingno packages (Supplemental Fig. 1). The amount of missingness was 0% or 0.14% in case of the ventilation score. The HFMSE score and CHOP-INTEND score showed 64.6% and 59.28% missing data points, respectively (Supplemental Fig. 1a). An in-depth analysis of missingness showed that, in line with the age-dependent use of each score, the CHOP-INTEND scores were missing when the HFMSE was used and vice-versa. To preserve the age-dependent meaning of these missing data points, we interpolated only the missing data points between two HFMSE and CHOP-INTEND scores for each patient. This reduced the amount of missing data from 64.6% to 46.04% and 59.28% to 33.96%, respectively (Supplemental Fig. 1b).

### Training and model evaluation

**Figure 1:**
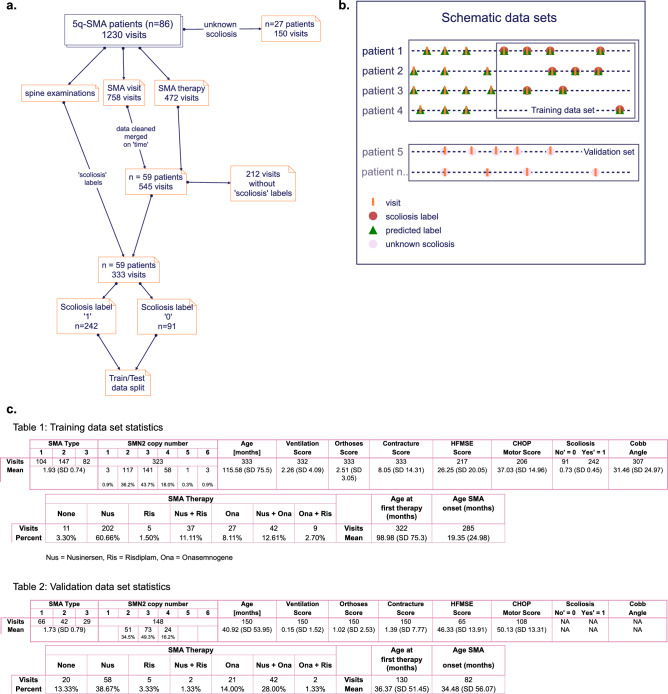
(**a**) Data processing flow in the predictive modeling process.1230 visits of 86 genetically confirmed SMA patients were cleaned and merged on time. The 333 visits were labeled with corresponding scoliosis labels from available spine examinations for supervised training of a RandomForestClassifier (RFC). (**b**) Schematic visualization of data subsets for training and validation. The model’s predictions were tested on visits without scoliosis labels and patient subsets where the scoliosis was unknown. (**c**) Table 1 summarizes the demographics and features in the training and testing data set used during model development. Table 2 summarizes the demographics of the validation data set used to validate the model after training.

To train an ML model, we used scikit-learn’s Random Forest Classifier and trained the model using 100 trees, and a maximum depth of 6. The model was trained using the following six features: ‘age at assessment’, ‘CHOP-INTEND score’, ‘HFMSE score’, ‘contractures score’, ‘ventilation score’, and ‘orthoses score’. Because of their feature randomness, bootstrapping and voting mechanisms, Random Forest Classifiers can handle moderate to severe class imbalance problems. The class distribution was moderately imbalanced in the training and testing data set. We used a *k* = 10 stratified Group-K-Fold cross-validation strategy^21^ to train and test the model. Using this cross-validation method, the train and test data sets were split to ensure that data from the same patient remained strictly separated in the train and test sets, respectively. The model’s performance was evaluated using accuracy, precision, recall, F1-score, and MCC. We plotted the average Received Operatic Characteristics Area Under the Curve (ROC AUC) curves and calculated the average AUC scores across ten folds and ten different random states. To validate our model, we tested the model on an unknown scoliosis data set (Fig. 1).

### Predicted probability of scoliosis versus Cobb angles

To further interrogate our model’s predictions, we plotted the predicted scoliosis probabilities and compared them with the actual scoliosis Cobb angles (Fig. 4). The saved model was then tested further on an ‘unknown scoliosis’ data subset consisting of 27 patients without scoliosis labels (Fig. 1). We then performed patient follow-up examinations to confirm the model’s prediction.

### Code availability

The code supporting the findings of this study is available on Zenodo at the following 10.5281/zenodo.11217091. This repository contains all source code of the data processing, machine learning model training, and testing.

### Approval by the Internal Review Board (IRB) and Ethics Commission

This study has been approved by Charité Universitätsmedizin Berlin Ethics Commission (EA2/178/21) and has been performed in accordance with the ethical standards laid down in the 1964 Declaration of Helsinki and its later amendments. Clinical patient data collected within this study has been collected with the informed consent from all subjects and/or their legal guardian(s).

## Results

The raw data set contained 1230 visits from 86 genetically confirmed 5q-SMA patients. The processed data frame consisted of 695 visits from 86 SMA patients. Scoliosis labels were available for 59 patients, resulting in a labeled train/test data set of 333 total visits. Patients without scoliosis labels (n = 27) corresponded with 150 visits (see also Fig. 1).

### Descriptive statistics of the training data set

Of 333 total visits from 59 SMA patients, 242 (72.7%) visits were labeled with ‘scoliosis’ and 91 (27.3%) visits with ‘no scoliosis’. SMA type 1 patients were represented with 104 (31.2%), SMA type 2 with 147 (44.1%) and SMA type 3 with 82 (24.6%) visits. There were 161 (48.35%) females and 172 (51.65%) males. The mean number of visits per patient was 5.64 (range 1–18). The mean age of patients was 115.58 months (SD 75.5), and the *SMN2* copy numbers ranged from 1 to 6. 3 patients (0.9%) had 1 *SMN2* copy number, 117 (36.2%) had 2 copy numbers, 141 (43.7%) had 3 copy numbers, 58 (18%) had 4 copy numbers, 1 (0.3%) had 5 copy numbers, and 3 (0.9) had 6 copy numbers (see Table 1 in Fig. 1c). The mean HFMSE score was 26.25 (SD 20.05) and the mean CHOP-INTEND score was 37.03 (SD 14.96), the mean Cobb angle was 31.46° (SD 24.97) (Table 1 in Fig. 1c). The mean age at SMA disease onset in the training data set was 19.35 months (SD 24.98) and the mean age at first SMA therapy was 98.98 months (SD 75.3) (Table 2 in Fig. 1c).

### Descriptive statistics of the validation data set

The validation data set contained 27 patients with unknown scoliosis labels and 150 visits. 66 (44%) of these visits represented SMA type 1, 42 (28%) represented SMA type 2, and 29 (19.3%) represented SMA type 3 patients. The mean age of patients in the validation data set was 40.92 months (SD 53.95), and the *SMN2* copy numbers ranged from 2 to 4. 2 patients had 51 (34.5%) *SMN2* copy numbers, 73 (49.3%) had 3 copy numbers and 24 (16.2%) had 4 copy numbers (Table 2 in Fig. 1c). The mean HFMSE score was 46.33 (SD 13.91), and the CHOP-INTEND score was 50.13 (SD 13.31). The mean age at first SMA disease onset was 34.48 months (SD 56.07) and the mean age at first SMA therapy was 36.37 months (SD 51.45) (Table 2 in Fig. 1c).

### Individual features alone are poor predictors of scoliosis

**Figure 2:**
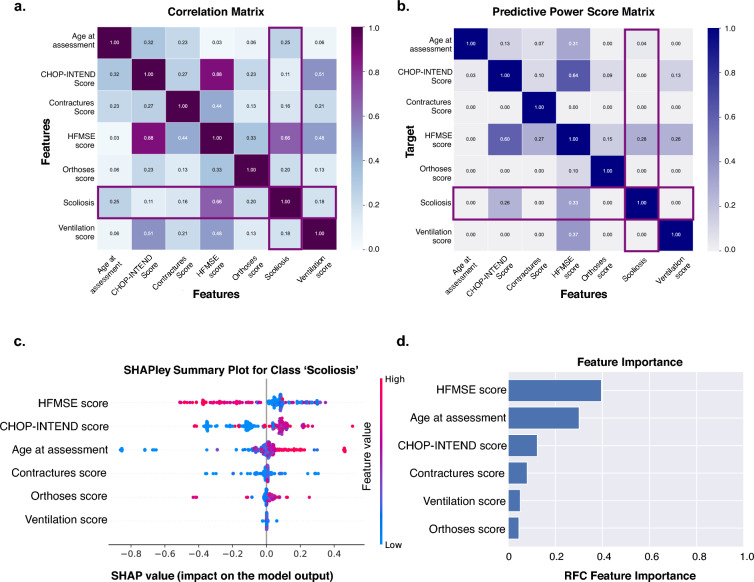
Selection of features ‘age at assessment’, ‘CHOP-INTEND score’, ‘HFMSE score’, ‘contractures score’, ‘orthoses score’, ‘ventilation score’ for training a RandomForestClassifier (RFC). (**a**) Correlation matrix (**b**) Predictive power score matrix (**c**) Feature importance from SHAPley values for the class 1, ‘scoliosis’ (**d**) Feature importance calculated with Gini Importance.

For univariate feature analysis we plotted the correlation coefficients and predictive power scores in a heatmap (Fig. 2a,b). When a patient transitioned from being evaluated using the CHOP-INTEND score to HFMSE score, both scores were strongly correlated (*r* = 0.88) (Fig. 2a). We observed weak correlation between the CHOP-INTEND and HFMSE score with the contractures score (*r* = 0.27 and 0.44, respectively). There was a moderate correlation between the ventilation score and the HFMSE and CHOP-INTEND score, (*r* = 0.48 and 0.51, respectively). The predictive power score showed that the CHOP-INTEND and HFMSE scores were moderately predictive of each other (PPS = 0.60 and 0.64). The HFMSE score, routinely used for patients 2 years and older^22^, predicted the age at assessment. Results of our multivariate feature analysis are shown in Fig. 2c,d. The SHAP value analysis showed that high values of HFMSE scores negatively impacted the model’s prediction for class 1 ‘scoliosis’. Increasing age at assessment positively impacted the prediction for class 1. In turn, low contracture scores negatively impacted class 1 prediction. The mean decrease impurity analysis over multiple folds showed relatively high feature importance for the HFMSE and CHOP-INTEND (Fig. 2). To test the predictive power of collective features over individual features, we compared ROC AUC curves (Supplemental Fig. 2), and observed that training on collective features improved model performance.

### Prediction of scoliosis based on collective clinical features

**Figure 3:**
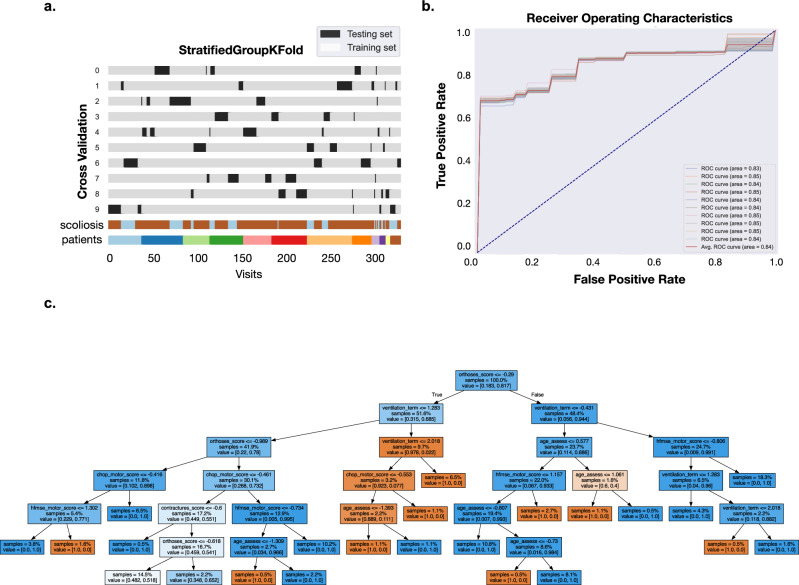
Training and performance evaluation of a binary Random Forest Classifier for scoliosis prediction. (**a**) Cross validation using StratifiedGroupKFold for ten folds (**b**) Average Receiver Operating Characteristics (ROC) for ten cross-validation folds of ten different random states (**c**) exemplary decision tree.

The model achieved a mean accuracy of 0.77 (SD 0.2) and an average ROC AUC of 0.85 (SD 0.17). For class 1 ‘scoliosis’ the average precision was 0.84 (SD 0.11), recall 0.89 (SD 0.22), F1-score of 0.85 (SD 0.17), respectively. The average Matthews correlation coefficient (MCC) was 0.40 (Supplemental Fig. 3b). The confusion matrices of ten stratified group K-folds are plotted in the Supplemental Fig. 1a. Detailed performance metrics for each stratified group K-fold can be found in Supplemental Fig. 3b. The results suggested variability across different stratified group K-folds. To get a more robust assessment of the model’s performance, we performed ten stratified group K-folds using different random states and calculated the average ROC AUC. The average ROC AUC across ten random states of grouped K-fold cross validation runs was 0.84 (SD 0.007) (Fig. 3).

### Predicted scoliosis probability

**Figure 4:**
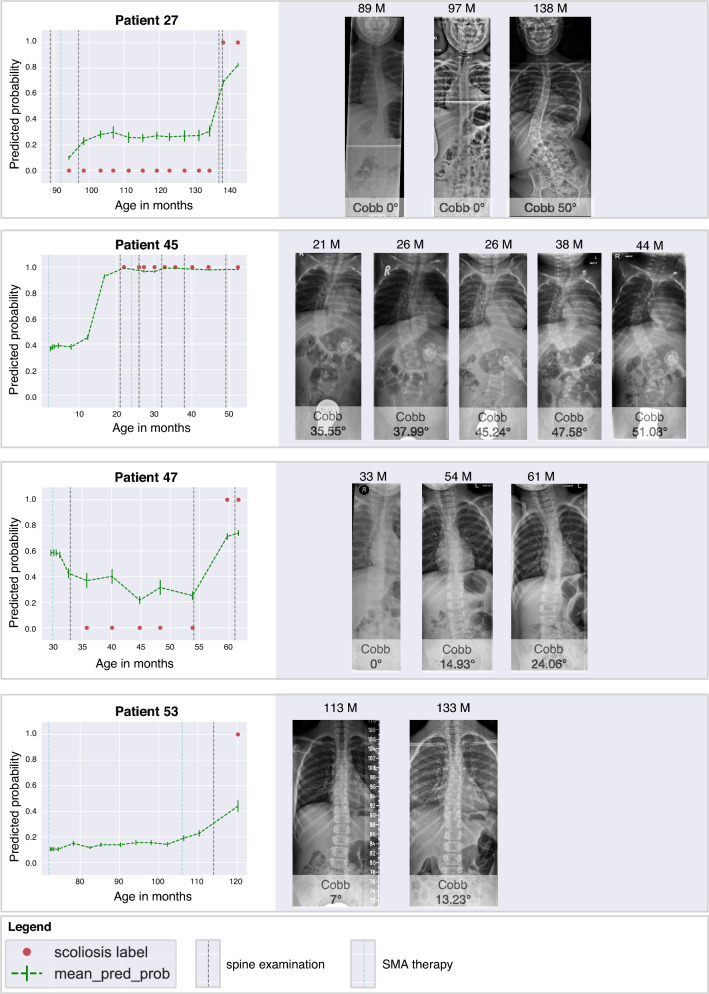
The model’s predicted scoliosis probability (green line) for patients 27, 45, 47, and 53 and their corresponding spine examinations or radiographs taken at the marked time points (grey line). The model was trained on data points corresponding with the scoliosis labels (red dots).

Using the learnt RFC model, we plotted the model’s predicted scoliosis probability for each patient. Here we could observe the changes in the model’s predicted probability of scoliosis over time, plotted as age of the patient in months (Fig. 4). Note that the Cobb angle was not used in the model’s training. When we plotted the model’s predicted probability of scoliosis and compared it with measured Cobb angles in spine radiographs, we could retrace the inflection point that marked the model’s predicted time point of scoliosis onset (Fig. 4).

### Model validation on ‘unknown scoliosis’ data set

**Figure 5:**
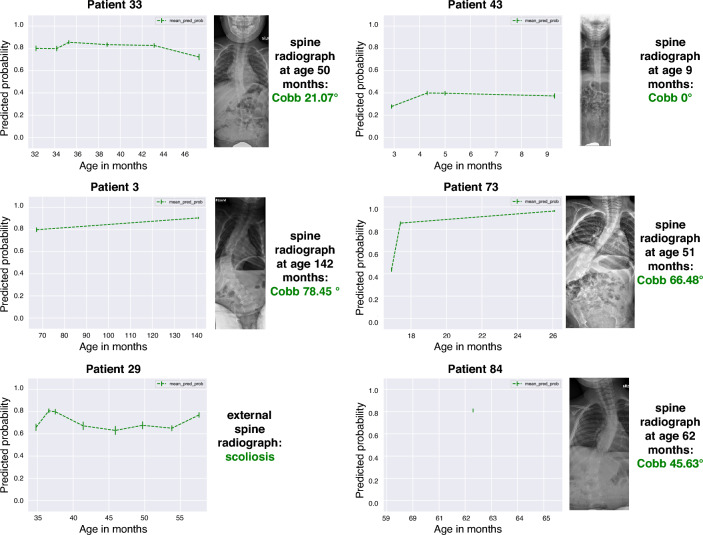
Model validation on the ‘unknown scoliosis’ data set. The predicted scoliosis probabilities for patients 3, 39, 33, 43, 73, and 84 are plotted on the left graph. The corresponding follow-up examinations of the spine are depicted on the right side of the graph.

We further tested our trained model on the unlabeled subset of the SMA data set and performed follow-up examinations of the patients to verify the model’s prediction. Some patients had either relocated or were deceased, so the predicted labels could not be verified for some. We were able to attain follow-up examinations from ten patients and the model had predicted the scoliosis label correctly for 9 of them. Figure 5 shows examples of the model’s predicted scoliosis probability and the results of our follow-up examinations.

## Discussion

SMA is one of the first monogenic neuromuscular disorders to receive gene therapy treatment. The efficacy of new SMA-therapies is changing the landscape of patient phenotypes. Early treatment administration improves outcomes ^23^, which is why SMA is now implemented in a growing number of newborn screening programs worldwide^2^. In addition, more effective modes for the administration of SMA therapies are currently being explored (e.g., in utero therapy)^24^. These advances in SMA treatment will create a dynamic SMA patient landscape, in which the anticipation of SMA disease progression and associated sequelae will guide decisions to provide the best standard of care. We developed an interpretable data-driven ML model that can predict scoliosis based on SMA progression markers, which can function as an assistive tool during interdisciplinary treatment evaluations. It is important to note that the current model has been trained to predict the likelihood that an SMA patient has scoliosis at a certain visit based on routinely assessed clinical features. This model has not been trained to forecast the presence of scoliosis at a future visit.

### Random forest classifier as an assistive tool

The aim of this study was to develop an assistive tool for physicians involved in the treatment of SMA patients that can help clinicians anticipate scoliosis onset. The model must be robust and interpretable for future adoption of the tool. Random Forest Classifiers have repeatedly demonstrated high accuracy and robustness in classification tasks. In addition, RFCs can capture nonlinear relationships between features and their target (here, ‘scoliosis’). By using different feature importance calculation methods, visualizing decision trees, and plotting the model’s predicted probabilities over time, the physician can interpret and interact with the model’s output and make decisions based on the presented predictions. How this technology will be used in the future will greatly depend on its integration with electronic health data collection and its adoption by physicians. Currently, clinical features of SMA patients are routinely assessed in the SMArtCare study. Such an algorithm could perform an automated scoliosis prediction based on routine feature inputs and suggest referrals to pediatric spine specialists, who can then use the model’s prediction with the child’s feature development to derive individualized treatment plans and schedule time points for reassessments and consultation.

### SMA data set statistics and representation

The training data set contained relatively balanced proportions of SMA types 1, 2, and 3 (31.23%, 44.14%, 24.62%, respectively). The gender distribution of 48.19% females to 51.81% males aligns with previously reported SMA statistics. The natural history of SMA is associated with scoliosis development in up to 60–95% of patients, depending on SMA types ^25,26^. The training data set contained 72.7% scoliosis, which was representative of the average prevalence of scoliosis in SMA^25^. Overall, the distribution between classes of 'scoliosis’ and ‘no scoliosis’ was moderately imbalanced, so we chose to train a Random Forest Classifier, which is known for its robustness even in severe class imbalances. Notably, most patients in this data set were born in Germany (72·0.09%), one of the first countries to approve SMA therapies and implement SMA in newborn screenings ^1^.

### Scoliosis labels and scoliosis prediction

The diagnosis of scoliosis is defined by a Cobb angle of > 10° (± 3°) on an anteroposterior spine radiograph, which may be debatable even among experts. Interestingly, some of the model’s predicted scoliosis probabilities reflected the uncertainty related to the diagnostic interval. For patient 53, the model predicted ‘scoliosis’ with a probability of only 41% (Fig. 4). When we compared the predicted probability with the corresponding spine radiograph, the measured Cobb angles were 7° and 13°, respectively, representing scoliosis onset. The current model was trained as a binary scoliosis classifier and therefore, cannot predict actual Cobb angles. Such a prediction model would require more complex models and larger data sets that include multiple spine radiographs taken in children with SMA at multiple time points. Ethically, this data set does not exist at the moment. Other approaches to address these obstacles could include data augmentation approaches in the future. However, it is remarkable that the model’s predicted scoliosis probability could reflect a diagnostic uncertainty in the physician’s spine radiograph. Future performance testing of the model should include an external validation data set. In addition, the model will likely improve when retrained with additional data.

### Feature engineering and feature selection

This model was designed to provide robust predictions regardless of baseline biomarkers, which is why we deliberately selected routinely assessed SMA progression markers for training. Excluding baseline SMA disease markers such as SMA type, *SMN2* copy numbers, motor milestones, first symptom onset, and SMA therapy, reduced our model’s performance metrics but, in our opinion, was optimized toward our model’s purpose and future application. Importantly, our focus on disease progression markers during model training is in line with the ongoing update of SMA classification^27^. To improve training on a rare disease data set, some features were engineered and presented as numeric using domain knowledge, which was based on the current evidence reported in the literature. Improved understanding of the impact of different features on SMA disease progression and scoliosis development, could further improve the feature engineering process and potentially, model performance in the future. Nevertheless, RFCs have demonstrated robustness in the context of different data types, including categorical and numerical.

### Limitations and *bias* in rare disease data sets

SMA is a rare disease. Data-driven models strongly depend on the data that they are trained on. Despite rigorous data preparation and feature selection to ensure meaningful data representation, potential biases in the training set may limit its current deployment. An external validation is needed in the next step. To the best of our knowledge, the data set used for the purpose of this study is one of the largest and most comprehensive SMA data sets to date. The robustness of the model should improve when it is retrained on a larger data set in the future. Potential solutions to mitigate current limitations may include using generative methods for data augmentation^28^. However, these methods require extensive evaluation before application for the purpose of clinical support tools. We were cautious in using such methods, as our current understanding of the new SMA patient landscape is still limited. Our predictive model, which utilizes SMA disease progression markers, could augment clinical decision-making in the future by offering quantified probabilities of scoliosis development, providing actionable insights that extend beyond the capabilities of traditional assessments. These insights would support targeted early intervention strategies and personalized care in neuromuscular disease management in the context of gene therapy.

## Conclusion

SMA will likely not be the last NMD to undergo a drug-induced alteration of the natural course of disease. New approaches to anticipate disease associated sequelae and progression will be needed to continuously provide the best standard of care. We demonstrate that rare disease data sets can be wrangled to build predictive ML models. These models harness the compounded observations that normally train a clinician’s intuition. Our trained model could predict scoliosis using selected disease progression markers, namely ‘age at assessment’, ‘HFMSE score’, ‘CHOP-INTEND score’, ‘ventilation score’, and ‘orthoses score’ and ‘contractures score’, and scoliosis prediction correlated with radiological Cobb angle measurements. More importantly, the model could help clinicians anticipate scoliosis in patients who were unseen during model training. Such ML models can function as assistive tools during interdisciplinary patient evaluations and augment expertise in an era of disease-modifying therapies. In addition, it has the potential to democratize expertise that is otherwise clustered at specialized centers. Future work entails giving access to and validating our model on a data set from an international clinic.

### Supplementary Information

Supplementary Information.

## Data Availability

The dataset generated during and analyzed during the current study are not publicly available, due to patient data protection. We will make the anonymized data subset used to train the final ML model in this study available through the corresponding author upon reasonable request. This will include data dictionaries. Specifically, the SMA data subset that underlie the results reported in this article (i.e. features used for training a Random Forest Classifier) after de-identification, i.e. anonymization (removal of record ID, age of the patient, dates of assessments, locations, genetic information). We will also provide our python code, including feature engineering code, written for this study. To protect the identity of patients and minimize the risk of re-identification in a rare disease data set, we would share the data only with investigators whose proposed use of the data has been approved by an independent review committee (“learned intermediary”) identified for this purpose. In concordance with our Clinical Trial Office’s Data Protection board, the data should not be made publicly available to avoid misuse, re-identification through data-pooling methods.

## Abbreviations

AUC: Area under the curve
CDC: Centers for disease control and prevention
CHOP-INTEND: Children’s Hospital of Philadelphia Infant Test of Neuromuscular Disorders
EHR: Eletronic health record
HFMSE: Hammersmith functional motor scale expanded
PACS: Picture archiving and communication system
RFC: Random forest classifier
ROC: Receiver operating characteristics
SHAP: Shapley additive exPlanations
SMA: Spinal muscular atrophy
