# Supplementary material for: An interpretable data-driven prediction model to anticipate scoliosis in spinal muscular atrophy in the era of (gene-) therapies
Source: Sci Rep. 2024 May 23;14:11838. doi: 10.1038/s41598-024-62720-w (PMC11116550; doi:10.1038/s41598-024-62720-w)
Supplement: Supplementary file 1 — Supplementary Information. [file 41598_2024_62720_MOESM1_ESM.docx]

## Appendix

### Feature Engineering

The detailed feature engineering process of the SMA scoliosis data set is described in another manuscript. The features used for training of our model included ‘orthosis’, ‘ventilation’, and ‘contractures’. The features were engineered as follows:

$$\text{orthosi}\text{s}_{\text{score}}= \sum_{i=1}^{10} {(2}^{i}\times\text{orthosis}_{i})^{\text{1/3 }}\text{orthosis}_{i}\in\left\{ 0, 1 \right\}$$

$$\text{ventilation}_{\text{term}}=(\frac{1}{\text{ventilation}_{\text{freq}}})\times\sum_{i=1}^{3} {(2}^{i}\times\text{ventilation}_{i}){\times\text{ventilator}_{\text{time}}}$$

$$\text{ventilation}_{i}\in\left\{ 0, 1 \right\}, \text{ventilation}_{\text{freq}}\in\left\{ 1, 2, 3 \right\}, \text{ventilator}_{\text{time}}\in\left\{ 1, 2, 3, 4 \right\}$$

$$\text{contracture}_{score}= \sum_{i=1}^{6} {(2}^{i}\times\text{loco}_{i})^{\text{contracture}\text{s}_{\text{limit}}/3}$$

$$\text{ loco}_{i}\in\left\{ 0, 1 \right\} , \text{contracture}\text{s}_{\text{limit}}\in\left\{ 1, 2, 3 \right\}$$
